# Discovery of Novel PI3Kδ Inhibitors Based on the p110δ Crystal Structure

**DOI:** 10.3390/molecules27196211

**Published:** 2022-09-21

**Authors:** Wenqing Jia, Shuyu Luo, Wennan Zhao, Weiren Xu, Yuxu Zhong, Dexin Kong

**Affiliations:** 1 Tianjin Key Laboratory on Technologies Enabling Development of Clinical Therapeutics and Diagnostics, School of Pharmaceutical Sciences, Tianjin Medical University, Tianjin 300070, China; 2 School of Stomatology, Hospital of Stomatology, Tianjin Medical University, Tianjin 300070, China; 3 Tianjin Institute of Pharmaceutical Research, Tianjin 300070, China; 4 State Key Laboratory of Toxicology and Medical Countermeasures, Beijing Institute of Pharmacology and Toxicology, Beijing 100850, China

**Keywords:** PI3Kδ, inhibitor, virtual screening, lead scaffold, cancer

## Abstract

PI3Kδ is a key mediator of B-cell receptor signaling and plays an important role in the pathogenesis of certain hematological malignancies, such as chronic lymphocytic leukemia. Idelalisib, which targets PI3Kδ specifically, is the first approved PI3K inhibitor for cancer therapy. Recently, we carried out virtual screening, cell-based assays, adapta kinase assays, and molecular dynamic analysis to discover novel PI3Kδ inhibitors and identified NSC348884 as a lead PI3Kδ inhibitor. NSC348884 had an excellent docking score, potent PI3Kδ-inhibitory activity, antitumor effects on various cancer cell lines, and a favorable binding mode with the active site of PI3Kδ. Moreover, through the structural modification of NSC348884, we further discovered comp#1, which forms H-bonds with both Val828 and Lys779 in the ATP binding pocket of PI3Kδ, with a more favorable conformation binding to PI3Kδ. In addition, we found that N^1^, N^1^, N^2^-trimethyl-N^2^-((6-methyl-1H-benzo[d]imidazol-2-yl) methyl) ethane-1,2-diamine might be a potential scaffold structure. Thus, the result of this study provides a far more efficient approach for discovering novel inhibitors targeting PI3Kδ.

## 1. Introduction

Phosphoinositide 3-kinases (PI3Ks) are a conserved family of kinases that phosphorylate phosphatidylinositol 4,5-trisphosphate (PIP2) to phosphatidylinositol 3,4,5-trisphosphate (PIP3), which acts as a second messenger to activate downstream signaling molecules. This phosphorylation process can be reversed by PTEN [1]. According to sequence homology, substrate selectivity, and differences in regulatory subunits, PI3Ks are divided into three classes. Among them, class I PI3Ks are further divided into IA (PI3Kα, β, and δ) and IB (PI3Kγ). PI3Kα and PI3Kβ are commonly expressed in all cells, while PI3Kδ and PI3Kγ are mainly expressed in hematopoietic cells and are closely related to immune system diseases [2]. Class II PI3Ks consist of three members: PI3KC2α, which is involved in endocytosis, vesicle trafficking, and mitosis; PI3KC2β, which acts in cell migration; and PI3KC2γ, known to activate Akt2 and liver glycogen storage [3]. The sole member of class III is named Vps34, whose functions are related to endocytosis, vesicular trafficking, and autophagy [4]. Among the three classes of PI3Ks, class I has been most widely studied and is therefore usually referred to as PI3K.

PI3Kδ, which consists of a catalytic subunit p110δ and a regulatory subunit p85, is a key mediator of B-cell receptor signaling and plays an important role in the pathogenesis of certain hematological malignancies, such as chronic lymphocytic leukemia (CLL) [5]. The proliferation of various hematologic tumor cells can be blocked by PI3Kδ inhibitors, and normal immune cells can survive in this process. Recent reports have demonstrated that the PI3Kδ isoform is associated with myeloid-cell-mediated immunosuppression in solid tumors [6], including head and neck squamous cell carcinoma (HNSCC) [7].

ZSTK474 is a pan class I PI3K inhibitor that shows the most potent inhibition against the PI3Kδ isoform [8,9]. PI3Kδ inhibitors include propeller-shaped and flat inhibitors. ZSTK474 is a flat inhibitor and mainly occupies the hinge region and affinity pocket of the ATP binding pocket of the catalytic subunit (p110δ) of PI3Kδ in a competitive way [10]. ATP can form hydrogen bonds with Val828 in the hinge region. The ATP-competitive PI3K inhibitors identified to date accept an H-bond from this valine residue. For ZSTK474, the oxygen of one of the morpholino groups acts as the hinge Val828 hydrogen bond acceptor. The benzimidazole group extends into the “affinity” pocket, where its nitrogen acts as a hydrogen bond acceptor for the primary amine of Lys779. Compared with the binding of ATP, ZSTK474 forms hydrogen bonds to the binding pocket, similar to those made by ATP. After exhibiting remarkable antitumor effect in preclinical studies, ZSTK474 has entered phase I/II clinical trials to evaluate its safety and therapeutic effect on patients with advanced solid malignancies (NCT01280487 and NCT01682473). However, acquired resistance after long-term treatment with ZSTK474 has been observed [11,12]. The crystal structure of the ZSTK474-PI3Kδ active site (2WXL) has been elucidated via X-ray analysis [13], which provides the basis for the discovery of novel PI3Kδ inhibitors. 

**Figure 1:**
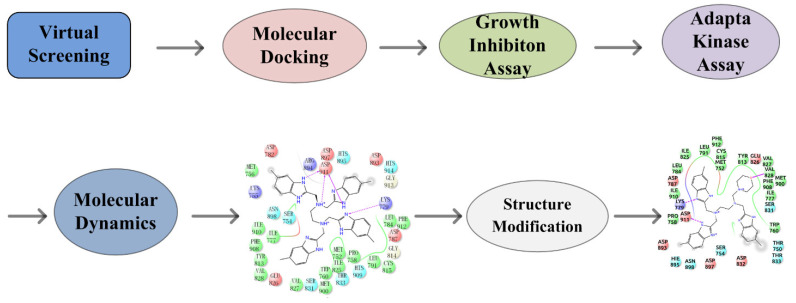
Flow chart of the discovery of the novel PI3Kδ inhibitor.

Idelalisib is the only PI3Kδ-specific inhibitor approved by the FDA and has shown solid antitumor efficacy clinically but was found to have various immune-mediated side effects, such as hepatotoxicity, pneumonitis, infection, and intestinal perforation [14,15]. To circumvent these problems, new PI3Kδ inhibitors are required. Virtual screening followed by experimental validation has been demonstrated to be a highly efficient approach for discovering molecular-targeted lead compounds. By utilizing such an approach, we found potential PI3Kδ inhibitors from the compound database established by our laboratory. In this work, we report the discovery of new PI3Kδ small-molecule inhibitors through virtual database screening in combination with an MTT assay, adapta kinase assay, and molecular dynamics simulation [16] (Figure 1).

## 2. Results and Discussion

### 2.1. Virtual Screening of Potential PI3Kδ Inhibitors

In this study, we used ZSTK474 as the positive drug to select compounds. Based on the results of HTVS and SP docking and by comparing docking scores and binding modes, seven commercially available compounds (Table 1) were selected from the database. The docking scores of all seven of these compounds are lower than that of ZSTK474.

### 2.2. Antiproliferative Effect of the Screened Compounds

**Figure 2:**
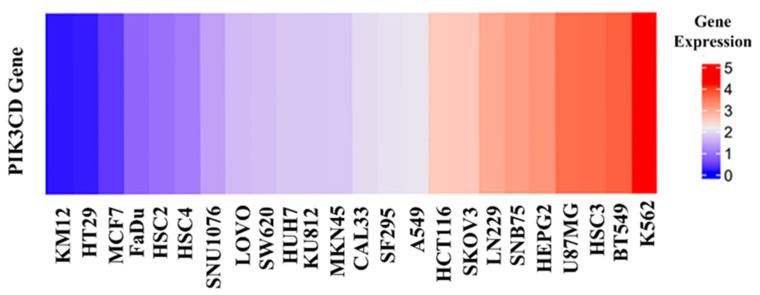
Gene expression levels in various cancer cell lines.

**Figure 3:**
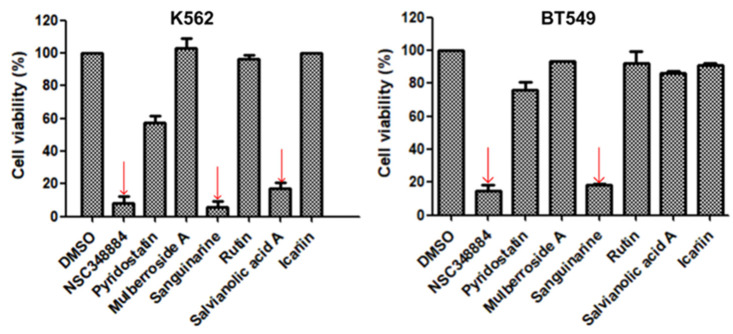
Effect of the selected compounds on cell viability of K562 and BT549 cells. The cells were treated with the seven compounds individually at 10 μM for 48 h, and the cell viability was analyzed via the MTT assay. Data are presented as the mean ± SD (*n* = 3).

As the above seven compounds were screened as potential PI3Kδ inhibitors, we tried to determine their antiproliferative activities on cancer cells possessing high PI3Kδ expression. We searched the Cancer Cell Line Encyclopedia database (CCLE) for the PIK3CD gene expression levels in established cell lines and cross-compared them with the cell lines available in our lab. As shown in Figure 2, the PIK3CD gene expression levels in human chronic myelogenous leukemia K562 and human breast ductal carcinoma BT549 cells were relatively higher than in other cells. We then treated K562 and BT549 cells with the above seven compounds at 10 μM for 48 h and determined the antiproliferative activity using the MTT assay. We found that three compounds (NSC348884, sanguinarine, and salvianolic acid A) inhibited the proliferation of K562 cells, with the viable cells reduced to 8.5%, 5.7%, and 17.3%, respectively, compared to the group without treatment. NSC348884 and sanguinarine exhibited similar antiproliferative effects in BT549 cells (14.7% and 18.4%, respectively), while salvianolic acid A only showed the minimum inhibition against BT549 cells (86.1% viable cells) (Figure 3).

### 2.3. PI3Kδ-Kinase-Inhibitory Activities of the Selected Compounds

**Figure 4:**
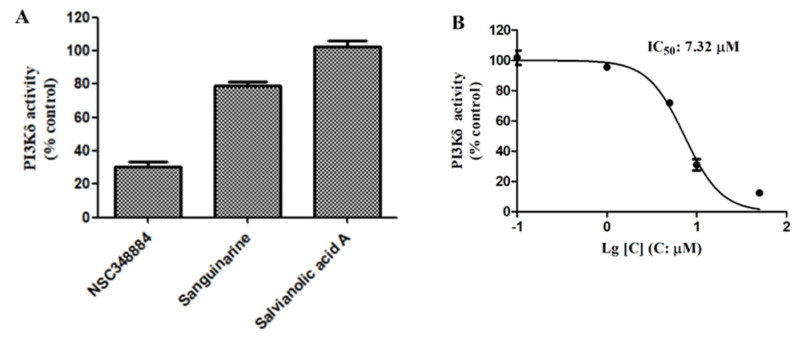
(**A**) Activities of 10 μM of NSC348884, sanguinarine, and salvianolic acid A on PI3Kδ. (**B**) NSC348884 inhibited PI3Kδ in a concentration-dependent manner with an IC_50_ of 7.32 μM. Data are presented as the mean ± SD (*n* = 3).

We then used the adapta kinase assay to determine the inhibition of the three compounds—NSC348884, sanguinarine, and salvianolic acid A—on the kinase activity of the recombinant PI3Kδ. After treatment with 10 μM of NSC348884 and sanguinarine, the activity of PI3Kδ was reduced to 30.15% and 79.27%, respectively. Salvianolic acid A at 10 μM, however, had no significant inhibitory effect on the kinase activity (Figure 4A). The IC_50_ value of NSC348884, the most potent inhibitor of the three compounds, was calculated to be 7.32 μM, as shown in Figure 4B.

### 2.4. The Mode of NSC348884 Binding to PI3Kδ

**Figure 5:**
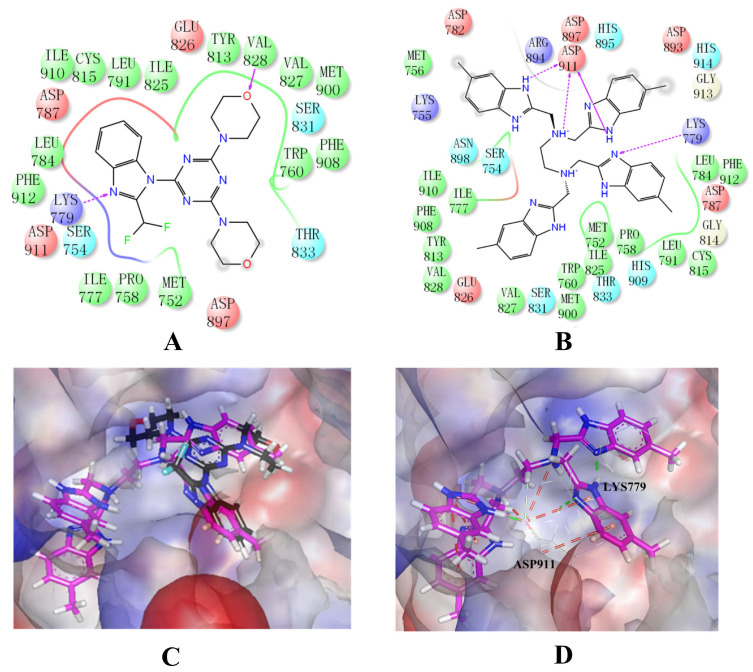
The predicted model of NSC348884 binding to the PI3Kδ pocket. (**A**) A 2D diagram of ZSTK474-PI3Kδ interactions in the PI3Kδ pocket. (**B**) A 2D diagram of NSC348884-PI3Kδ interactions in the PI3Kδ pocket. (**C**) NSC348884 (purple)/ZSTK474 (black)-PI3Kδ pocket. (**D**) NSC348884 (purple)-PI3Kδ pocket.

Binding mode studies are of great significance for the discovery of more potent inhibitors based on the mechanism of the known inhibitors and PI3Kδ. The morpholine oxygen atom of ZSTK474 was reported to form a key H-bond with the hinge region Val828, and the N3 of benzimidazole generated another H-bond with Lys779, consistent with our result (Figure 5A). Accordingly, we studied the mode of NSC348884 binding to PI3Kδ. As shown in Figure 5B, NSC348884 bound to PI3Kδ and formed multiple H-bonds with nearby residues, including Lys779 and Asp911. NSC348884 maintained the H-bond with the key amino acid Lys779, as found for ZSTK474. Asp911 formed H-bonds with two imidazole rings and amino groups of NSC348884. Compared with ZSTK474, NSC348884 had more H-bond interactions, which might be responsible for the lower docking score (ZSTK474-PI3Kδ: −9.49 kcal/mol; NSC348884-δ: −9.69 kcal/mol). Figure 5C shows the superposition of ZSTK474 and NSC348884 in the binding pocket, and the binding mode of NSC348884 is indicated in Figure 5D. The benzimidazole group in NSC348884 basically overlaps with that of ZSTK474. This phenomenon also reasonably explains why both compounds formed the same H-bond with Lys779.

### 2.5. Molecular Dynamics Simulations of NSC348884-PI3Kδ

**Figure 6:**
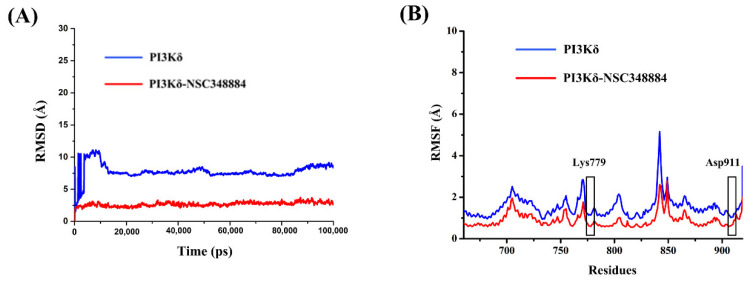
(**A**) The RMSD changes in NSC348884-PI3Kδ complexes and PI3Kδ during 100 ns simulations. (**B**) The RMSF changes in NSC348884-PI3Kδ complexes and PI3Kδ during 100 ns simulations. The boxes indicate the fluctuations of key residues (Lys779 and Asp911).

Molecular dynamics simulation is the investigation of the internal motions of a macromolecules as a function of time. The root mean square deviation (RMSD) is used to examine the stability and convergence of backbone Cα atoms relative to the initial structure [17]. For PI3Kδ without ligands, before 12 ns, the RMSD values experienced a rapid rise from 0 to 10.92 Å and then gradually tended to reach an equilibrium status (Figure 6A). In the NSC348884-PI3Kδ model, although the RMSD also increased initially, the value stabilized below 3.72 Å after 5 ns and then remained balanced (Figure 6A). In addition, the average RMSD value of NSC348884-PI3Kδ was 2.68 Å, which was lower than that of the PI3Kδ-without ligand system (7.76 Å) (Figure 6A). This shows that both systems reached the equilibrium condition after a short simulation, and NSC348884-PI3Kδ was more stable. The root mean square fluctuation (RMSF) was used to calculate the average value of all atomic fluctuations [18]. Figure 6B shows the RMSF maps of PI3Kδ-ligand complexes and PI3Kδ during simulations. Overall, the fluctuation value for NSC348884-PI3Kδ was lower than the PI3Kδ-without ligand. The fluctuation values of Lys779 and Asp911 in NSC348884-PI3Kδ were lower than the value in PI3Kδ only, which suggests that the binding of NSC348884 to PI3Kδ made the whole system more stable.

**Figure 7:**
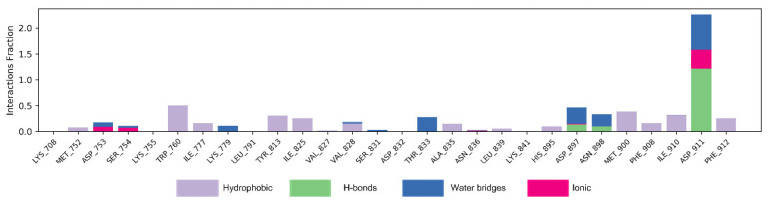
The bar charts of NSC348884-PI3Kδ interactions. The legend for each color section is given.

Figure 7 shows the interaction mode of NSC348884 and PI3Kδ in the whole simulation process in the form of a histogram. A value above 1.0 indicates that there are multiple interactions between amino acid residues and ligands. H-bonds play important roles in the drug specificity, metabolic characteristics, absorption parameters, and blood–brain barrier permeability of drug candidates. For NSC348884-Asp911, the H-bond interaction fraction is 1.214 (>1.0), indicating that Asp911 binds with the ligand via multiple interactions of H-bonds, and the H-bonds are relatively stable (Figure 7 and Table 2). Lys779 can also form an H-bond with a ligand but mainly forms H-bonds with ligands indirectly through water molecules. In addition, during the simulation of 100 ns, Asp897 and Asn898 also form H-bonds with the ligand, and the H-bond occupancy are 13.5% and 9.2%, respectively. The hydrophobicity of Trp760 and Met900 also contributes to the stability of the whole system, and the occupancy of hydrophobic contacts is 50.3% and 38.6%, respectively.

### 2.6. Structural Modification of NSC348884

**Figure 8:**
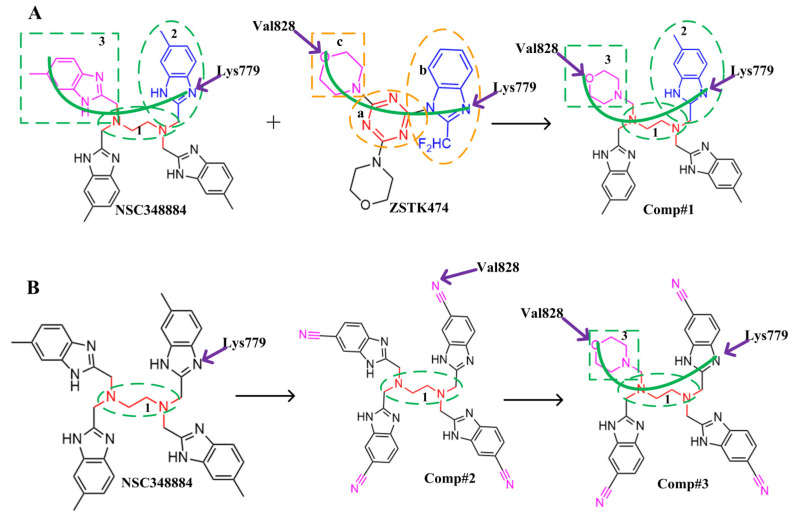
Schematic diagram of the structural modification of NSC348884. (**A**) Design of Comp#1 via structural modification of NSC348884; (**B**) design of Comp#2 and Comp#3 via structural modification of NSC348884. a: triazine; b: benzimidazole; c: morpholino; 1: tetramethylethane-1,2-diamine; 2: 6-methyl-benzimidazole.

**Figure 9:**
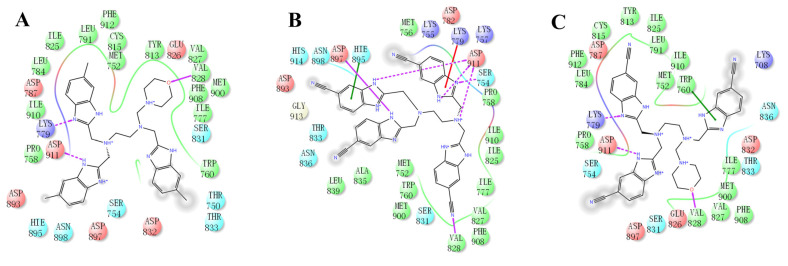
The predicted binding modes of comp#1 (**A**), comp#2 (**B**), and comp#3 (**C**) to the PI3Kδ pocket. The 2D diagrams of comp#1/2/3-PI3Kδ interactions in the PI3Kδ pocket are indicated.

**Figure 10:**
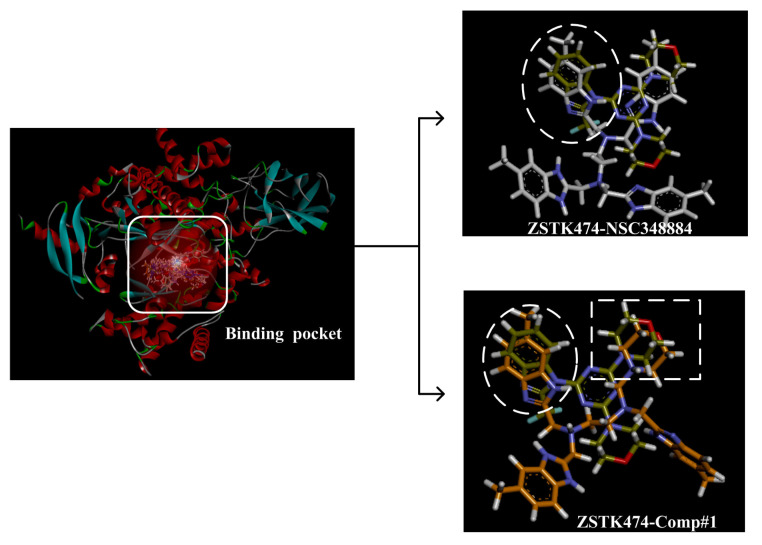
Superposition of ZSTK474/NSC348884 and ZSTK474/Comp#1. Grayish green: ZSTK474; white: NSC348884; orange: Comp#1.

The above results suggest that NSC348884 might be a potential PI3Kδ inhibitor. This finding provides a novel scaffold structure for the discovery of novel PI3Kδ inhibitors. We further modified NSC348884 to improve its affinity. The designed structural modifications are shown in Figure 8. We analyzed and compared the structures of NSC348884 and ZSTK474, as well as the binding modes between the compounds and PI3Kδ. To modify NSC348883, we tried the following approaches:

(1) Both ZSTK474 and NSC348884 contain a benzimidazole group, and this group forms an H-bond with the key amino acid Lys779. As the main scaffold, the benzimidazole should be retained during modification (Figure 8A). 

(2) ZSTK474 is a triazine derivative. The benzimidazole group extends into the “affinity” pocket, where its nitrogen acts as an H-bond acceptor for Lys779. The oxygen of the morpholino groups forms an H-bond with Val828 as the hinge H-bond acceptor. To improve the affinity of NSC348884 with the target, we introduced a group that can form an H-bond with Val828. We used tetramethylethane-1,2-diamine and 6-methyl-benzimidazole in NSC348884 as the scaffold structure. By analyzing the position of benzimidazole N3 and the oxygen atom on the morpholine ring in ZSTK474, we introduced a morpholine group to NSC348884 to obtain comp#1. A molecular docking study indicated that the docking score of comp#1 was −10.52 kcal/mol, lower than that of NSC348884 (−9.69 Kcal/mol). As expected, comp#1 formed H-bonds with Lys779 and Val828, and the H-bond with Asp911 remained (Figure 8A and Figure 9A). 

(3) The direct introduction of H-bond acceptors could form H-bonds with Val828 on 6-methyl-benzimidazole, as exhibited by Comp#2 (Figure 8B and Figure 9B). The docking results showed the introduction of a nitrogen atom enabled comp#2 to form an H-bond with Val828, with a docking score lower than that of NSC348884. However, the H-bond at Lys779 disappeared. To achieve the dual purpose, we introduced a morpholine ring to comp#2 and obtained comp#3. The docking results showed that comp#3 could form H-bonds with Val828 and Lys779 (Figure 9C), with a docking score of −10.43 Kcal/mol, lower than that of comp#2. 

(4) By comparing comp#1 with comp#3, we found that the mode of binding between comp#1 and PI3Kδ was more similar to ZSTK474. In both compounds, Lys779 and Val828 could form H-bonds with nitrogen atoms in benzimidazole and oxygen atoms in the morpholine ring, respectively. The docking score of comp#1 was also lower than that of comp#3. Therefore, we chose comp#1 for further evaluation. The reason for the formation of H-bonds between comp#1 and Lys779/Val828 could also be explained by the superposition diagram of ZSTK474 and NSC348884/Comp#1 in the binding pocket. As shown in Figure 10, the benzimidazole basically overlapped, and the distance between the oxygen atoms on morpholine rings was close in the ZSTK474/NSC348884 superposition diagram (Figure 10). The degree of superposition determines the strength of H-bonds between compounds and key amino acids. Therefore, comp#1 has better affinity to PI3Kδ. Detailed information on these three compounds is shown in Table 3.

## 3. Materials and Methods

### 3.1. Virtual Screening and Molecular Docking Study

The crystal structures of PI3Kδ (ID: 2WXL with a resolution of 1.99 Å) were acquired from RCSB Protein Data Bank (PDB, www.rcsb.org, accessed on 6 September 2020). The ligand of PI3Kδ, ZSTK474, was used as a positive control. The compound database containing 16,000 compounds was collated by our group, which mainly consists of the following compounds: FDA-approved drugs, drugs in the clinical research phase, and compounds with biological activity in preclinical studies. Both natural products and synthetic compounds are included in this database.

ZSTK474, a pan PI3K inhibitor with the most potent inhibitory activity towards the PI3Kδ isoform, is under clinical trial evaluation for the therapy of advanced solid malignancies as an oral drug [12,19]. Since the crystal structure of ZSTK474-PI3Kδ has been elucidated [20], ZSTK474 can be used as a reference ligand for the discovery of novel PI3Kδ inhibitors. Molecular docking was applied using GLIDE software from the Schrödinger suite 2009 Software Package [21]. The proteins downloaded from PDB have missing residues. Protein repair uses the prime module of Schrodinger. Prime can be used to create homology models for use in simulations and to repair protein structures. Protein structures were prepared using the PrepWizard module. The optimization of the H-bond was performed using an exhaustive sampling option [22]. Under the Impref module, the OPLS_2005 force field was determined [23]. The active site was defined as an enclosing box at the centroid of the workspace ligand as selected in the receptor folder. The box size was determined by dock ligands with lengths ≤ 8.5 Å. Finally, under the application module, a docking grid was obtained.

The selected ligands from the compound library containing diverse chemical datasets were prepared in the Ligprep module. The possible ionization states were generated at a target pH of 7.0 ± 2.0 using Ionizer. Generate tautomers was selected by default. The tautomerizer generates up to 8 tautomers per ligand. At most, 32 stereoisomers per ligand were obtained with the option of “Retain specifified chiralities (vary other chiral centers)”. [24]. The prepared molecules and PI3Kδ protein were then subjected to high-throughput virtual screening (HTVS) [25]. Based on the HTVS results, the GLIDE SP (standard precision) module [26,27] was subsequently used to obtain new PI3Kδ inhibitors. The interaction mechanisms between virtual hits and the receptor were evaluated at the molecular docking stage. 

### 3.2. Cell Lines and Culture Conditions

The human chronic myelogenous leukemia K562 and human breast ductal carcinoma BT549 cell lines were purchased from Cell Resource Center, Peking Union Medical College (Beijing, China). Cells were cultured in Roswell Park Memorial Institute (RPMI) 1640 medium (HyClone, Cramlington, UK) (for K562) and Dulbecco’s Modified Eagle Medium (DMEM, HyClone, Cramlington, UK) (for BT549), supplemented with 10% (*v*/*v*) fetal bovine serum (FBS, Biological Industries, Kibbutz Beit-Haemek, Israel), 100 U/mL of penicillin, and 10 μg/mL of streptomycin at 37 °C in a humidified atmosphere containing 5% CO_2_ [28].

### 3.3. Cell Proliferation Assay

As previously reported [29,30], K562 cells and BT549 cells were plated at 2 × 10^4^ cells/well in 96-well plates. Cells were treated with or without compounds for 48 h. After that, 20 μL of MTT was added into 96-well plates and incubated for another 4 h at 37 °C. The culture medium was discarded, and 150 μL of DMSO was added into 96-well plates. The light absorbance was detected at a wavelength of 490 nm by using a microplate reader (iMark, Bio Rad, Hercules, CA, USA). Data were analyzed by using the GraphPad Prism 5 software.

### 3.4. Adapta Kinase Assay

To study the PI3K-inhibitory activity of compounds, the kinase activity of PI3Kδ in the presence or absence of the compounds was measured using the adapta kinase assay [31]. The kinase reaction was manipulated in 10 μL total volume of the reaction buffer containing diluted compounds, PI3Kδ kinase, 2 mM DTT, 0.1 mM substrate, and 0.02 mM ATP, in a 384-well plate for 1 h at 28 ± 1 °C. Then, 5 μL of buffer containing 30 mM EDTA, 6 nM antibody, and 12 nM tracer was added and incubated for 0.5 h at room temperature. The kinase activity remaining in each well was calculated according to the formula: kinase activity (% control) = (sample-minus enzyme control)/(plus enzyme control–minus enzyme control) × 100. For the plus enzyme control, PI3Kδ kinase was incubated with its substrate PIP2 and ATP in the absence of the test compound (sample), and for the minus enzyme control, the substrate PIP2 was incubated with ATP in the absence of the kinase and the test compound. Data were analyzed using the GraphPad Prism 5 software.

### 3.5. Molecular Dynamics Simulations Assay

The molecular dynamics (MD) simulations were performed by using Desmond v4.3 software to analyze the stability and conformational changes in ligands, proteins, and protein–ligand complexes through trajectory charts. Briefly, a successful MD process consists of the following six parts: preparing proteins, building a model system, relaxing the model system, running the simulation, viewing the trajectory, and analyzing the results.

We prepared protein using protein preparation wizard. To make the molecular dynamics more realistic, we added a solvent environment to mimic the real environment of humans. In this process, the water model and box shape were set to SPC and orthorhombic, respectively, in order to accommodate the water molecules in the system. We applied “Buffer” as the method to calculate the box size within the dimensions of 10 Å × 10 Å × 10 Å. Moreover, counter ions were added to electrically neutralize the system. Default salt (NaCl) with the approximately physiological concentration of 0.15 M was placed in the simulation box to set the ionic strength. Ultimately, the biological system consisting of proteins, ligands, explicit solvent (water molecules), counter ions, and salt was set up. Then, the minimization process was performed to relax the system into local energy minimization because the model system built using the system builder panel was not optimal. The system was minimized using a hybrid method of the steepest descent and the limited-memory Broyden–Fletcher–Goldfarb–Shanno algorithms. After minimization, this model system was submitted to MD simulation steps using the Desmond v4.3 package with the OPLS_2005 force field. The relaxation times in the coupled thermostat and barostat methods were fixed to 1 and 2 ps, respectively. We used a cut-off radius of 9.0 Å to handle short-range coulombic interactions and a smooth particle mesh Ewald (PME) method with a 10^−9^ tolerance to calculate the long-range coulombic interactions. The SHAKE algorithm was used to satisfy the hydrogen bond geometry constraints during simulation. The integration time model for ‘bonded’, ‘near’, and ‘far’ options were set to 2, 2, and 6 fs, respectively.

Before performing the real simulations, a series of short molecular simulation (the first seven stages) steps were performed to relax the model system. In detail, stage 1 mainly aimed to detect the system type and traits. In stage 2, the restraints were put on solute-heavy atoms with small time steps (100 ps, 10 K) under Brownian dynamics NVT condition. With restraints on solute-heavy atoms (12 ps, 10 K) under NVT and NPT conditions, the next two stages were performed. In the sixth stage after the solvate pocket had been skipped in stage 5, restraints were sequentially exerted on solute-heavy atoms for 12 ps (NPT). Next, the process of no restraints (24 ps) was performed with NPT in stage 7. Once the system was equilibrated after the above-mentioned steps, the 100 ns simulation task (stage 8) was conducted at 300 K and 1.01325 bar of pressure without any restrictions. After the simulation, the analysis was run using simulation interaction diagram and simulation event analysis panels [21].

## 4. Conclusions

While dozens of PI3K inhibitors, including PI3Kδ inhibitors, are in clinical evaluation, only five PI3K inhibitors have been approved. As the first approved PI3K inhibitor, idelalisib was approved by the FDA in 2014 for the treatment of recurrent CLL in combination with retuximab, or as a single drug to cure recurrent follicular B cell non-Hodgkin’s lymphoma (FL) and recurrent small lymphocytic lymphoma (SLL). Notably, idelalisib is the only approve PI3Kδ-specific inhibitor. The delayed progress in the development of other PI3Kδ inhibitors is due to failure to achieve the desired efficacy, probably because of limited bioavailability, unpredictable toxicity, or acquired resistance. Given these impediments, there remains an urgent need to develop novel PI3Kδ inhibitors.

In our study, we aimed to find more effective PI3Kδ inhibitors by using virtual screening, based on the interaction mechanism existing between PI3Kδ and ZSTK474. Of the seven candidate compounds tested, NSC348884, sanguinarine, and salvianolic acid A showed remarkable inhibitory effects on the growth of tumor cells. The adapta kinase assay was performed to verify the direct target of the compounds. The results demonstrate that NSC348884 is a PI3Kδ inhibitor. Then, SP and MD analyses were used to evaluate the binding stability of NSC348884-PI3Kδ. The docking score of PI3Kδ-NSC348884 was lower than that of PI3Kδ-ZSTK474, and the compound formed H-bonds with Lys779 in the binding pocket.

Through analyses of the structure, molecular docking, and molecular dynamics of NSC348884/ZSTK474-PI3Kδ, we found that NSC348884-PI3Kδ binding is stable, and this binding excludes H-bond forming with Val828. Therefore, we hypothesized that the structure could be modified to form H-bonds with two key amino acids by introducing a morpholine ring into NSC348884. The result showed that benzimidazole in comp#1/ZSTK474 basically overlaps, and the distance between oxygen atoms on the two morpholine rings is also very close. H-bonds in comp#1 and ZSTK474 with Lys779 and Val828 formed on the same nitrogen atom and oxygen atom, and the docking score of comp#1 was lower than that of ZSTK474, suggesting that comp#1 might be a more effective PI3Kδ inhibitor.

In summary, our study demonstrated that NSC348884 is a novel PI3Kδ inhibitor, and the structure modification of NSC348884 generates comp#1, which was expected to have higher binding affinity. The PI3Kδ-inhibitory activity as well as antitumor effects will be determined in the future. This work provides a rapid approach to find novel PI3Kδ inhibitors, and N^1^, N^1^, N^2^-trimethyl-N^2^-((6-methyl-1H-benzo[d]imidazol-2-yl) methyl) ethane-1,2-diamine might be a potential scaffold structure for PI3Kδ inhibitor development.

## Figures and Tables

**Table 1:** The structures and docking results of molecules.

Name	Structure	Docking Score (kcal/mol)
Rutin	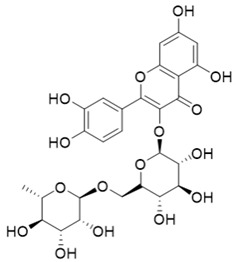	−11.14
Pyridostatin	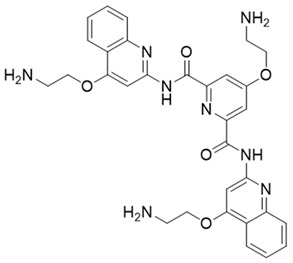	−10.69
Icariin	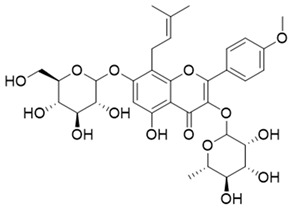	−10.35
Mulberroside A	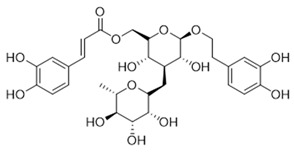	−10.12
Sanguinarine	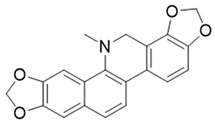	−10.10
NSC348884	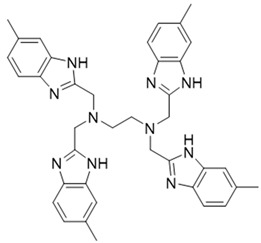	−9.69
Salvianolic acid A	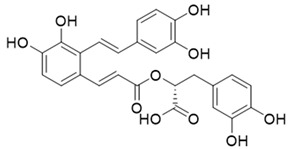	−9.61
ZSTK474	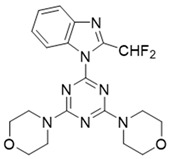	−9.49

**Table 2:** The occupancy of the protein–ligand interactions.

Ligand	Amino Acid	Occupancy of the Protein–Ligand Interactions (%)			
		H-Bonds	Hydrophobic Contacts	Ionic Interactions	Water Bridges
NSC348884	Trp760		50.3%		
	Ile777		15.9%		
	Tyr813		30.6%		
	Ile825		26.1%		
	Val828		15.4%		2.8%
	Ala835		14.9%		
	His895		10.2%		
	Met900		38.6%		
	Ile910		31.8%		0.1%
	Phe912		26%		
	Thr833				27.3%
	Lys779	0.3%			10.9%
	Asp897	13.5%		1.5%	31.7%
	Asn898	9.2%		0.1%	23.8%
	Asp911	>100% (1.214)		36.6%	68.1%

Note: For NSC348884-Asp911, the interaction fraction is 1.214 (>1.0), indicating Asp911 makes multiple interactions of H-bonds with the ligand.

**Table 3:** Structures and docking scores of the designed compounds.

Name	Structure	Docking Score (kcal/mol)
Comp#1	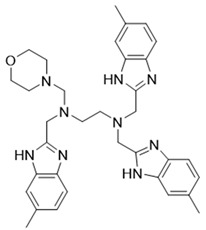	−10.52
Comp#2	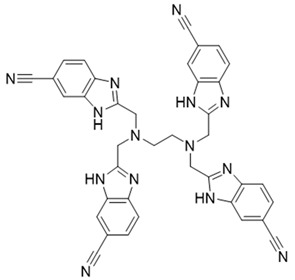	−10.28
Comp#3	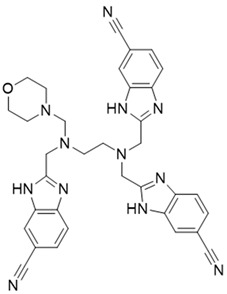	−10.43

## Data Availability

The data presented in this study are available on request from the corresponding authors.
